# PPARγ coactivator-1α (PGC-1α) protects neuroblastoma cells against amyloid-beta (Aβ) induced cell death and neuroinflammation via NF-κB pathway

**DOI:** 10.1186/s12868-017-0387-7

**Published:** 2017-09-25

**Authors:** Yuqin Zhang, Changchun Chen, Yanliu Jiang, Shupei Wang, Xiaoyu Wu, Kai Wang

**Affiliations:** 1 Department of Neurology, The First Affiliated Hospital of Anhui Medical University, Hefei, 230032 Anhui China; 2 Department of Neurology, The Second People’s Hospital of Anhui Province, Hefei, 230011 Anhui China

**Keywords:** PGC-1α, Aβ, Neuronal death, Neuroinflammation, NF-κB pathway

## Abstract

**Background:**

Alzheimer’s disease is characterized by the accumulation of amyloid beta (Aβ) and the formation of neurofibrillary tangles. Aβ is the main constituent of senile plaques and is largely involved in neuronal death and neuroinflammation. Peroxisome proliferator-activated receptor gamma coactivator-1 alpha (PGC-1α) is one of the main transcriptional coactivator and has been related to many fields such as energy metabolism, cardiovascular disease, neurodegenerative disorders, and so on.

**Results:**

Treatment with Aβ1–42 reduced the expression of PGC-1α in both protein and RNA levels of neuroblastoma N2a cells. Aβ1–42 induced a robust activation of cleaved caspase-3 while PGC-1α suppressed this activation and protected N2a cells from Aβ-induced cell death. Overexpression of PGC-1α significantly reduced the level of main proinflammatory cytokines. In addition, PGC-1α inhibited the transportation of NF-κB p65 from cytoplasm to nucleus and IκBα degradation induced by Aβ1–42.

**Conclusion:**

Our results have demonstrated that PGC-1α can protect neuroblastoma cells against Aβ-induced neuronal death and neuroinflammation. Moreover, this neuroprotective effect of PGC-1α is regulated through NF-κB pathway. Taken together, our work provides evidence that PGC-1α could be beneficial in targeting Aβ neurotoxicity.

## Background

Alzheimer’s disease (AD) is one of the most prevalent neurodegenerative disorders and is characterized by neuronal degeneration and death in the central nervous system [1, 2]. At present, AD affects about 40 million people and has become a healthcare challenge worldwide. Two predominant pathological features of AD brains are extracellular aggregation of amyloid-beta (Aβ) and formation of intracellular neurofibrillary tangles [3]. As a major factor in the development of AD, Aβ is the main constituent of senile plaques and is largely involved in neuronal death and neuroinflammation [4–7]. However, the molecular mechanisms underlying Aβ induced neurotoxicity and neuroinflammation still remain unclear [8–12].

It has been reported that Aβ-induced neuroinflammation contributes to the progress of neurodegeneration and neuronal death in Alzheimer’s disease [13]. Under the inflammatory condition, glia cells and some immune cells are activated directly or indirectly by increased levels of potent proinflammatory cytokines such as IL-1α, IL-1β, IL-6 and TNF-α, etc [14, 15]. Moreover, some signaling pathways are initiated during this process including nuclear factor-kappa B (NF-κB) pathway. The transcription factor NF-κB is crucial for many biological processes such as inflammation, differentiation, apoptosis, neuronal survival, and so on. In the central nervous system, NF-κB signaling pathway has drawn attention because of its functions in synaptic plasticity, neuronal survival, and neurotransmission [16–19]. For example, the activation of NF-κB was detected in AD brains and is a key step in Aβ-induced neurotoxicity [20]. Therefore, the regulation of the NF-κB pathway could be a perspective target in the treatment of AD.

The peroxisome proliferator-activated receptor gamma (PPARγ) is an isoform of PPARs that regulates numerous biological processes like metabolism of glucose and lipids, inflammation, cellular differentiation, and proliferation [21]. This nuclear receptor is regulated by other genes called transcriptional coactivators. Peroxisome proliferator-activated receptor gamma coactivator-1 alpha (PGC-1α) is one of the main transcriptional coactivators and has been involved in many processes such as energy metabolism, cardiovascular disease, neurodegenerative disorders, and so on. Several studies have reported that overexpression of PGC-1α can reduce the production of Aβ [22, 23]. Thus, whether PGC-1α can protect neurons from Aβ-induced cytotoxicity has not been well understood.

In this study, we investigate first, whether Aβ treatment can induce the change of PGC-1α expression in neuroblastoma cell line; second, whether PGC-1α can protect neuroblastoma cells from Aβ-induced neuronal death and neuroinflammation; and third, whether the neuroprotective characteristic of PGC-1α to Aβ is mediated by the NF-κB pathway. Notably, we determined that PGC-1α protects the neuroblastoma cells from Aβ neurotoxicity by inhibiting the NF-κB pathway. These results provide us with a novel and potential therapeutic strategy for AD.

## Methods

### Antibodies and reagents

The following antibodies were used in this study: anti-PGC-1α (1:1000, #4259), anti-cleaved caspase-3 (1:1000, #9664), anti-NF-κB p65 (1:1000, #8242), anti-phospho-IκBα (1:1000, #2859), anti-Lamin B1 (1:1000, #13435), anti-IκBα (1:1000, #9242), and β-actin (1:3000, #3700) were purchased from Cell Signaling Technology, Inc. (Danvers, MA, USA). Aβ1–42 fragments were purchased from Sigma-Aldrich Inc. (St. Louis, MO, USA). A stock solution of the peptides were dissolved in ultrapure water at 1 mM. Aβ1–42 was then diluted with the experimental solution to 25 μM and vortexed thoroughly. This Aβ1–42 solution was used within 5 min of preparation. With this preparation procedure, the Aβ1–42 was largely present as stable oligomers.

### Cell culture and transfection

We used mouse neuroblastoma N2a cells from American Type Culture collection (Manassas, VA, USA) in our experiments. Cells were cultured in Dulbecco’s Modified Eagle medium (DMEM) with 10% Fetal Bovine Serum (Biomeda, Fostercity, CA, USA) and 1% penicillin at 37 °C in a humidified incubator with 5% CO_2_. All other cell culture regents were obtained from Gibco (Carlsbad, CA, USA). Transfection with empty plasmid or pcDNA4 myc PGC-1α (PGC-1α plasmid) (Addgene, Cambridge, MA, USA) were performed according to the manufacturer’s instruction with Lipofectamine 2000 from Invitrogen (Carlsbad, CA, USA). Cells were used for the following experiments 48 h after transfection.

### Western blot analysis

After different treatments, cells were lysed by sonication in CelLytic MT buffer (Sigma, St. Louis, MO, USA) and proteins were extracted. 50 μg protein samples were separated on a denaturing 10% SDS-PAGE gel, and transferred to polyvinylidene fluoride membranes. The membrane was then blocked in PBST with 5% non-fat milk at room temperature for 1 h, followed by incubation with specific primary antibodies at 4 °C overnight. Blots were incubated with appropriate secondary antibodies at room temperature for 1 h the next day. The signals were detected by ECL detection kit. Blots were analyzed by ImageJ software.

### Quantitative real-time PCR

mRNA levels of PGC-1α were determined by quantitative real-time polymerase chain reaction (qRT PCR). Total RNA was extracted from 1 × 10^7^ with TRIzol (Invitrogen, Carlsbad, CA, USA) and cDNA from different samples were amplified with specific primers for human PGC-1α [5′-GGC AGA AGG CAA TTG AAG AG-3′ (forward) and 5′-TCA AAA CGG TCC CTC AGT TC-3′ (reverse)] and GAPDH [5′-ATCAGCAATGCCTCCTGCAC-3′ (forward) and 5′-CGTCAAAGGTGGAGGAGTGG-3′ (reverse)] Data were normalized to GAPDH expression and the control group was set as 1.

### TUNEL assay

TUNEL assay was performed to measure the apoptotic neuroblastoma cells after treatment with an in situ cell death detection kit (Roche Diagnostics, Indianapolis, IN, USA). According to the manufacturer’s instruction, cells were TUNEL-stained, followed by counterstaining with DAPI. TUNEL positive cells were detected and counted with a confocal microscope.

### Cell viability assay

1 × 10^5^ N2a cells were plated in each well of a 96-well plate with 100 μl medium. After treatment, cell viability was assessed by the Cell Counting Kit-8 (Dojindo, Japan) according to the manufacturer’s instructions. We prepare control wells without cells and subtract the background absorbance of the control wells.

### Enzyme-linked immunosorbent assay (ELISA)

After treatment, levels of IL-1β and TNF-α in the culture supernatant were measured by IL-1β and TNF-α ELISA kits (BD Bioscience, San Diego, CA, USA) according to the manufacturer’s instructions.

### Statistical analysis

All data was analyzed using unpaired Student’s *t* tests or two-way ANOVA with Bonferroni post-tests (GraphPad Software) and represented as the mean ± SEM of at least three independent experiments. *p* values were calculated with the appropriate statistical tests using GraphPad Prism software 7.0. A significant difference was considered to be present at *p* < 0.05.

## Results

### Treatment with Aβ1–42 reduces the expression of PGC-1α in neuroblastoma N2a cells

**Fig. 1:**
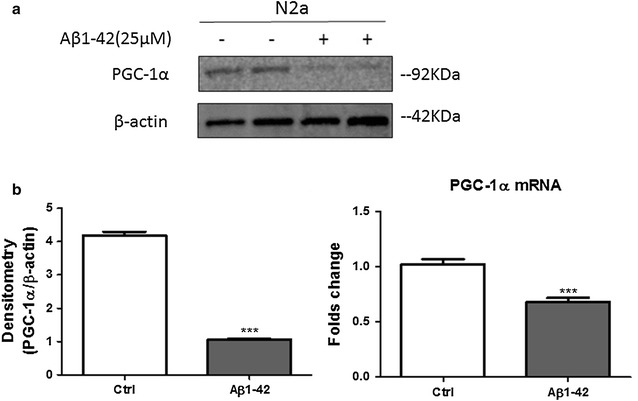
Aβ1–42 treatment affects PGC-1α expression of neuroblastoma N2a cells. **a** N2a cells were treated with or without Aβ1–42 (25 μM) for 6 h. Cell lysates were harvested and subjected to Western blot analysis. Protein level of PGC-1α was detected and β-actin was used as loading control. **b** Quantification of PGC-1α mRNA levels in N2a cells treated with Aβ1–42 (25 μM) by qRT-PCR. Results are expressed as mean ± SEM (unpaired Student’s *t*-tests) and representative for three independent experiments. ***p < 0.001 compared to control group

To evaluate whether Aβ1–42 treatment will affect the PGC-1α level, we first treated N2a cells with or without Aβ1–42 fragments (25 μM) for 6 h. Western blot analysis showed that the protein level of PGC-1α was significantly decreased compared the non-treatment group (Fig. 1a). Further quantification of PGC-1α mRNA levels in N2a cells was performed after a 6 h Aβ1–42 treatment. Treatment with Aβ1–42 resulted in a robust decrease in PGC-1α transcription (Fig. 1b).

### PGC-1α attenuates Aβ1–42 induced N2a cell death

**Fig. 2:**
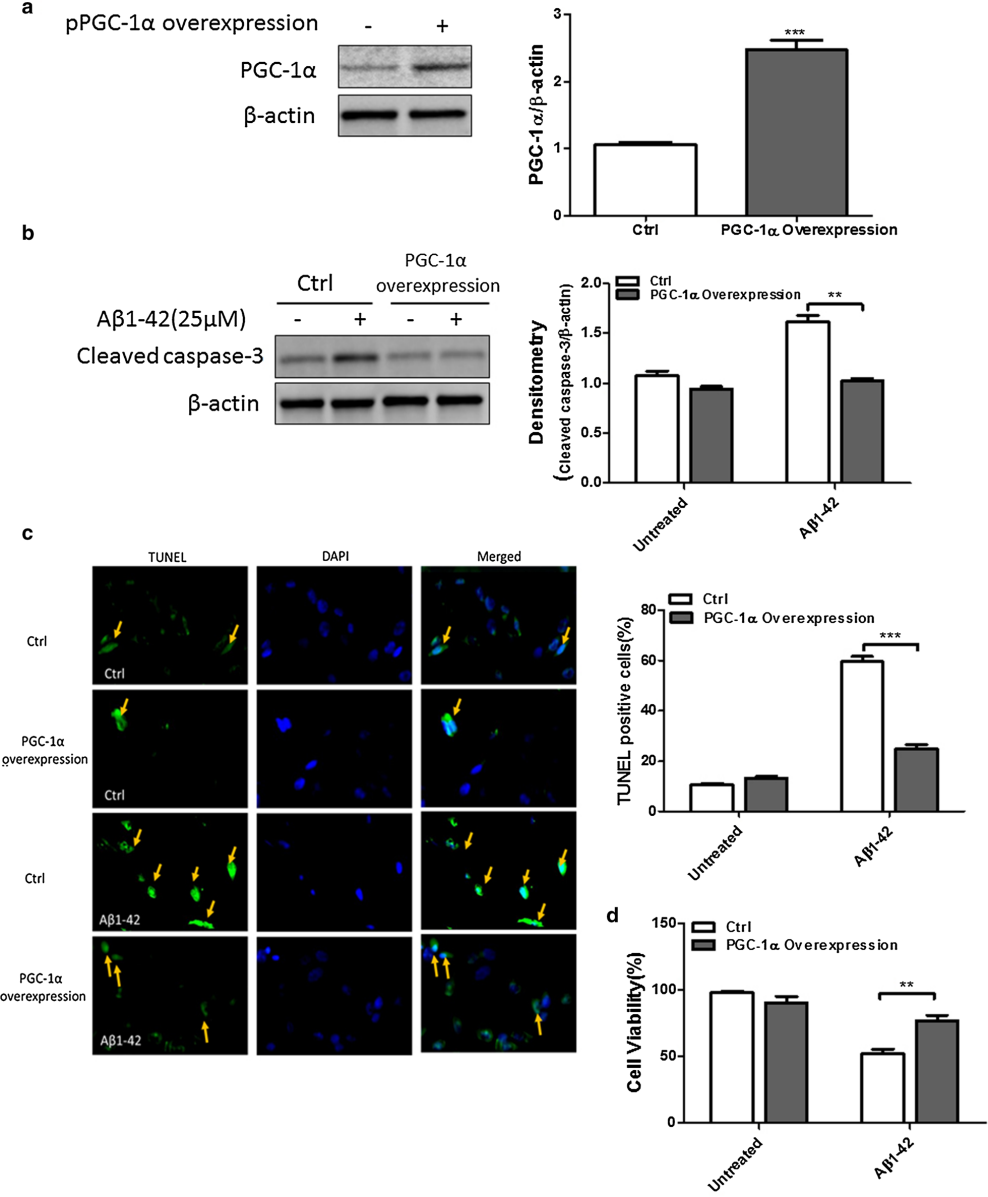
Effects of PGC-1α on Aβ1–42 induced N2a cell death. **a** N2a cells were transfected with an emoty vector or PGC-1α overexpression plasmid followed by Western blot analysis of PGC-1α level. **b** N2a cells were transfected with an empty vector or PGC-1α overexpression plasmid and then treated with or without Aβ1–42 (25 μM) for 6 h. Cell lysates were harvested and immunoblotted for cleaved caspase-3. β-actin was used as loading control. **c** Representative pictures of TUNEL assay and Quantification of apoptotic cells by fluorescent TUNEL assay. **d** Cell viability was measured by CCK8. Results shown are demonstrated as mean ± SEM (two-way ANOVA with Bonferroni post-tests) and representative for three independent experiments. **p < 0.01, ***p < 0.001 compared to control group

Neuron loss is one of the main pathological features that occur in AD. In this study, we want to determine if PGC-1α can protect neuroblastoma N2a cells against neuron cell death. We successfully overexpressed PGC-1α by transfecting N2a cells with pcDNA4 myc PGC-1α (PGC-1α plasmid) (Fig. 2a). N2a cells which were transfected with an empty vector or PGC-1α plasmid were then treated with or without Aβ1–42 (25 μM) for 6 h. We examined the expression of cleaved caspase-3, an apoptotic marker, using immunoblotting analysis. Aβ1–42 induced a robust activation of cleaved caspase-3 while PGC-1α suppressed this activation (Fig. 2b). TUNEL assay was then performed to verify the neuronal apoptosis. After Aβ1–42 treatment, the number of TUNEL positive cells was significantly reduced in the PGC-1α overexpression group compared to the control group (Fig. 2c). Moreover, we examined the cell viability by CCK8 after Aβ1–42 treatment and found that PGC-1α inhibited the reduction of cell viability induced by Aβ1–42 (Fig. 2d). These results suggest the protective role of PGC-1α in Aβ1–42 induced N2a cell death.

### PGC-1α prevents neuroinflammation induced by Aβ1–42

**Fig. 3:**
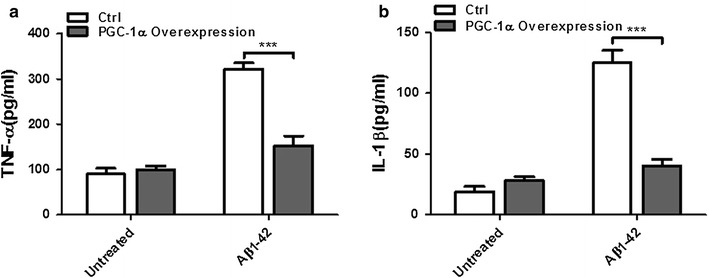
Effects of PGC-1α on Aβ1–42 induced neuroinflammation. N2a cells transfected with an empty vector or PGC-1α overexpression plasmid were treated with or without Aβ1–42 (25 μM) for 6 h. Culture media was collected and subjected to ELISA for **a** TNF-α and **b** IL-1β. Results shown represent the mean ± SEM (two-way ANOVA with Bonferroni post-tests) for three independent experiments. ***p < 0.001 compared to control group

The AD mice model exhibits a pronounced neuroinflammatory feature in the brain which will cause the later development of AD. N2a cells transfected with an empty vector or a PGC-1α overexpression plasmid were challenged with or without Aβ1–42 (25 μM) for 6 h. Two main proinflammatory cytokines, TNF-α (Fig. 3a) and IL-1β (Fig. 3b), were secreted into culture media then examined by ELISA. These two cytokines were significantly reduced in the PGC-1α overexpression group compared with the control group.

### PGC-1α inhibits Aβ1–42 induced N2a cell death via NF-κB pathway

**Fig. 4:**
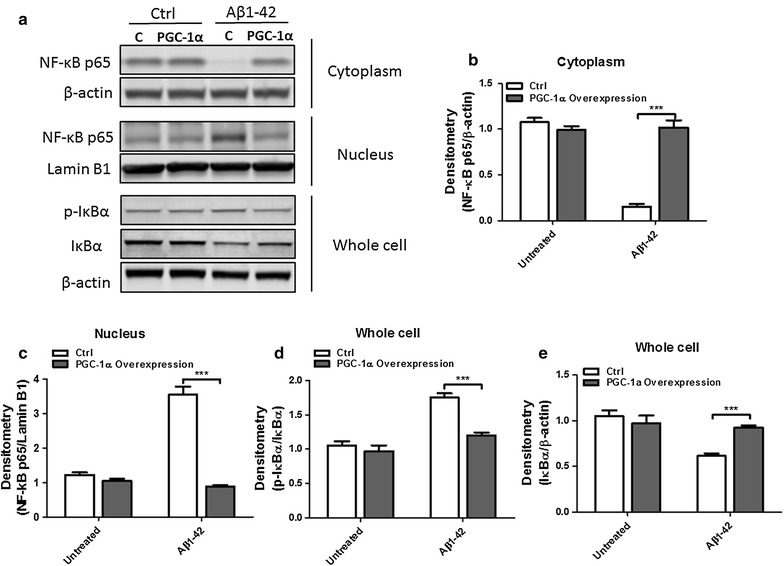
PGC-1α inhibits NF-κB pathway induced by Aβ1–42 in N2a cells. N2a cells transfected with an empty vector or PGC-1α overexpression plasmid were treated with or without Aβ1–42 (25 μM) for 6 h. **a** Cytoplasmic extracts, nuclear extracts and whole cell lysates were prepared and subjected to Western blot analysis for NF-κB p65, Lamin B1, p-IκBα, IκBα, and β-actin was used as loading control. The levels of **b** Cytoplasmic NF-κB p65, **c** nuclear NF-κB p65, **d** p-IκBα, and **e** IκBα were quantified by densitometry. Results shown are representative as mean ± SEM (two-way ANOVA with Bonferroni post-tests) for three independent experiments. ***p < 0.001 compared to control group

The NF-κB pathway plays a critical role in many biological progresses in the nervous system one of which is regulation of inflammatory responses. In the AD mouse model, it has been shown that the NF-κB pathway is activated by Aβ which leads to subsequent neuron loss and neuroinflammation. To test whether PGC-1α can inhibit the activation of the NF-κB pathway induced by Aβ1–42, we transfected N2a cells with an empty vector or a PGC-1α overexpression plasmid followed by a treatment with or without Aβ1–42 (25 μM) for 6 h. Proteins from cytoplasm (Fig. 4b), nucleus (Fig. 4c), and whole cell (Fig. 4d) were extracted and subjected to Western blot analysis for NF-κB p65, Lamin B1, p-IκBα, and IκBα. PGC-1α significantly inhibits the transportation of NF-κB p65 from cytoplasm to nucleus induced by Aβ1–42. Moreover, IκBα degradation induced by Aβ1–42 was also attenuated by PGC-1α. Taken together, these results suggest that PGC-1α attenuated the activation of NF-κB pathway induced by Aβ1–42 in N2a cells.

## Discussion

AD is a prevalent neurodegenerative diseases with cognitive function and memory impairment in the elderly. Several hypotheses of AD pathogenesis have been proposed in current AD studies such as Aβ hypothesis, tau hypothesis and the cholinergic hypothesis [24]. Recently Aβ hypotheses are most commonly used in this field. Molecular mechanisms of Aβ-induced neurotoxicity have been investigated for the last two decades to understand the pathology process of AD. However, current therapeutics has very minimal impact to target the underlying cause of this disease [25–27]. In this study, our aim is to determine a coactivator of PPARγ, PGC-1α, and to test whether this coactivator can protect neuroblastoma cells from Aβ-induced neurotoxicity.

We first measured the levels of PGC-1α after the treatment with Aβ1–42. Both protein and mRNA levels of PGC-1α are reduced with Aβ challenge. This data is consistent with the conclusion of some studies based on animal model and clinical trial. These studies showed that PGC-1α expression decrease in AD brains as the results of Aβ pathology [23].

We then test how PGC-1α affects the Aβ-induced neuronal death and neuroinflammation. After treating with Aβ1–42 for 6 h, PGC-1α prevents neuroblastoma cells from death and largely reduces the level of IL-1β and TNF-α. Inflammation in the brain is a complicated network and is affected by many proinflammatory cytokines, including IL-6, IL-1β, TNF-α, IL-17, and so on [7, 28, 29]. Among these cytokines, IL-1β and TNF-α are key regulators involved in AD pathogenesis. Some studies have shown that their levels and activities were increased in the AD brains. Moreover, both of them are implicated to have protective effects to Aβ [30–34].

The transcription factor NF-κB plays numerous roles in the nervous system, including its function in synaptic processes, neuroprotection, neural stem cell proliferation, neurotransmission, and so on. In addition, its involvement in brain inflammation is very important for us to understand the protective role of PGC-1α. In AD brain, many mediators can activate the NF-κB pathway, including Aβ accumulation, reactive oxygen species, and numerous proinflammatory cytokines [35–37]. During this activation, the IκBα will be phosphorylated and then degraded. NF-κB will be activated and enters into the nucleus where it induces the gene transcription for some inflammatory mediators. In this study, we determined that PGC-1α inhibits the activation and translocation of NF-κB from cytoplasm to nucleus. Meanwhile, the degradation of IκBα is also inhibited by PGC-1α. These results implicate that NF-κB pathway is essential in the protective processes of PGC-1α against Aβ.

## Conclusion

Our work demonstrates the neuroprotective function of PGC-1α to Aβ-induced neurotoxicity and the biological mechanisms underlying this process. These results provide us with promising evidence that PGC-1α could have a therapeutic effect to combat the neuronal death and neuroinflammation associated with AD.

## Abbreviations

AD: Alzheimer’s disease
PGC-1α: peroxisome proliferator-activated receptor gamma coactivator-1 alpha
Aβ: amyloid-beta
NF-κB: nuclear factor-kappa B
CCK8: Cell Counting Kit-8
IL-1β: interleukin-1 beta
TNF-α: tumor necrosis factor-alpha
